# Supplementary material for: Two New Mitogenomes of Bibionidae and Their Comparison within the Infraorder Bibionomorpha (Diptera)
Source: Genes (Basel). 2023 Jul 21;14(7):1485. doi: 10.3390/genes14071485 (PMC10378959; doi:10.3390/genes14071485)
Supplement: Supplementary file 1 [file genes-14-01485-s001.zip › Additional file 2 Table S2.pdf]

Table S2 Composition and skewness of 20 species of mtgenomes in the Bibionomorpha.

Superfamily	Species	A%	T%	G%	C%	A+T%	AT-Skew	GC-Skew
Bibionoidea	*Penthetria simplioipes*	40.20	39.40	8.00	12.30	79.60	0.0101	-0.2118
	Piecia hardyi	40.00	39.90	8.20	11.80	79.90	0.0013	-0.1800
	Cramptonomyia spenceri	38.90	37.20	9.10	14.80	76.10	0.0223	-0.2385
Anisopodoidea	*Sylvicola fenestralis*	37.90	38.40	10.10	13.50	76.30	-0.0066	-0.1441
Scatopsoidea	*Coboldia fuscipes*	39.50	36.70	9.50	14.30	76.20	0.0380	-0.1990
Sciaroidea	*Mayetiola destructor*	44.60	39.50	7.10	8.80	84.10	0.0606	-0.1069
	Orseolia oryzae	47.40	38.30	6.20	8.10	85.70	0.1062	-0.1329
	Rhopalomyia pomum	44.60	40.60	6.50	8.30	85.20	0.0469	-0.1216
	Sciara ruficauda	40.10	38.80	8.70	12.40	78.90	0.0165	-0.1754
	Pseudolycoriella sp.	39.10	39.90	9.30	11.60	79.00	-0.0101	-0.1100
	Pnyxia scabiei	41.40	35.80	7.70	15.10	77.20	0.0725	-0.3246
	Trichosia lengersdorfi	40.90	38.50	8.00	12.60	79.40	0.0302	-0.2233
	Neoempheria proxima	39.90	38.80	8.80	12.40	78.70	0.0140	-0.1698
	Epicypta sp.	38.60	40.80	9.10	11.10	79.40	-0.0280	-0.1020
	Epicypta xiphothorna	38.70	39.90	8.90	12.40	78.70	-0.0152	-0.1643
	Azana sp.	42.50	37.70	7.10	12.70	80.20	0.0501	-0.2828
	Acnemia nitidicollis	42.20	38.30	7.80	11.80	80.50	0.0484	-0.2041
	Allodia sp.	38.00	37.80	10.30	13.90	75.90	0.0026	-0.1488
	Allodia anglofennica	38.30	37.80	10.10	13.90	76.00	0.0066	-0.1583
	Arachnocampa flava	39.60	42.40	7.30	10.60	82.00	-0.0341	-0.1843
