# Supplementary material for: Two New Mitogenomes of Bibionidae and Their Comparison within the Infraorder Bibionomorpha (Diptera)
Source: Genes (Basel). 2023 Jul 21;14(7):1485. doi: 10.3390/genes14071485 (PMC10378959; doi:10.3390/genes14071485)
Supplement: Supplementary file 1 [file genes-14-01485-s001.zip › Additional file 4 Figure S1.pdf]

Figure S1. Predicted secondary structures for 22 tRNAs in *Penthetria simplioipes* (A), and *Plecia hardyi* (B) mtgenomes. Nucleotides highlighted in red indicate mismatch.

A

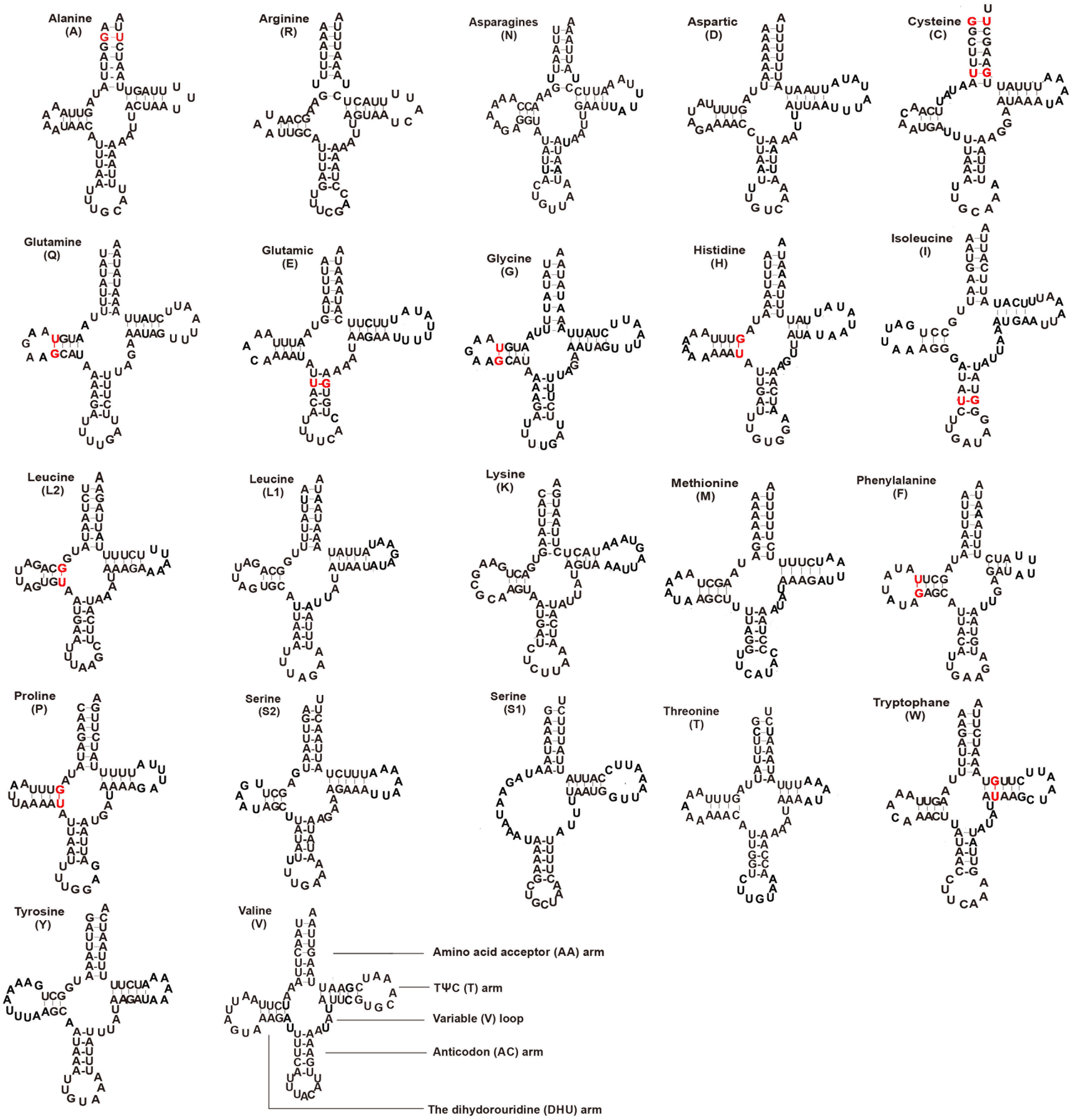

B

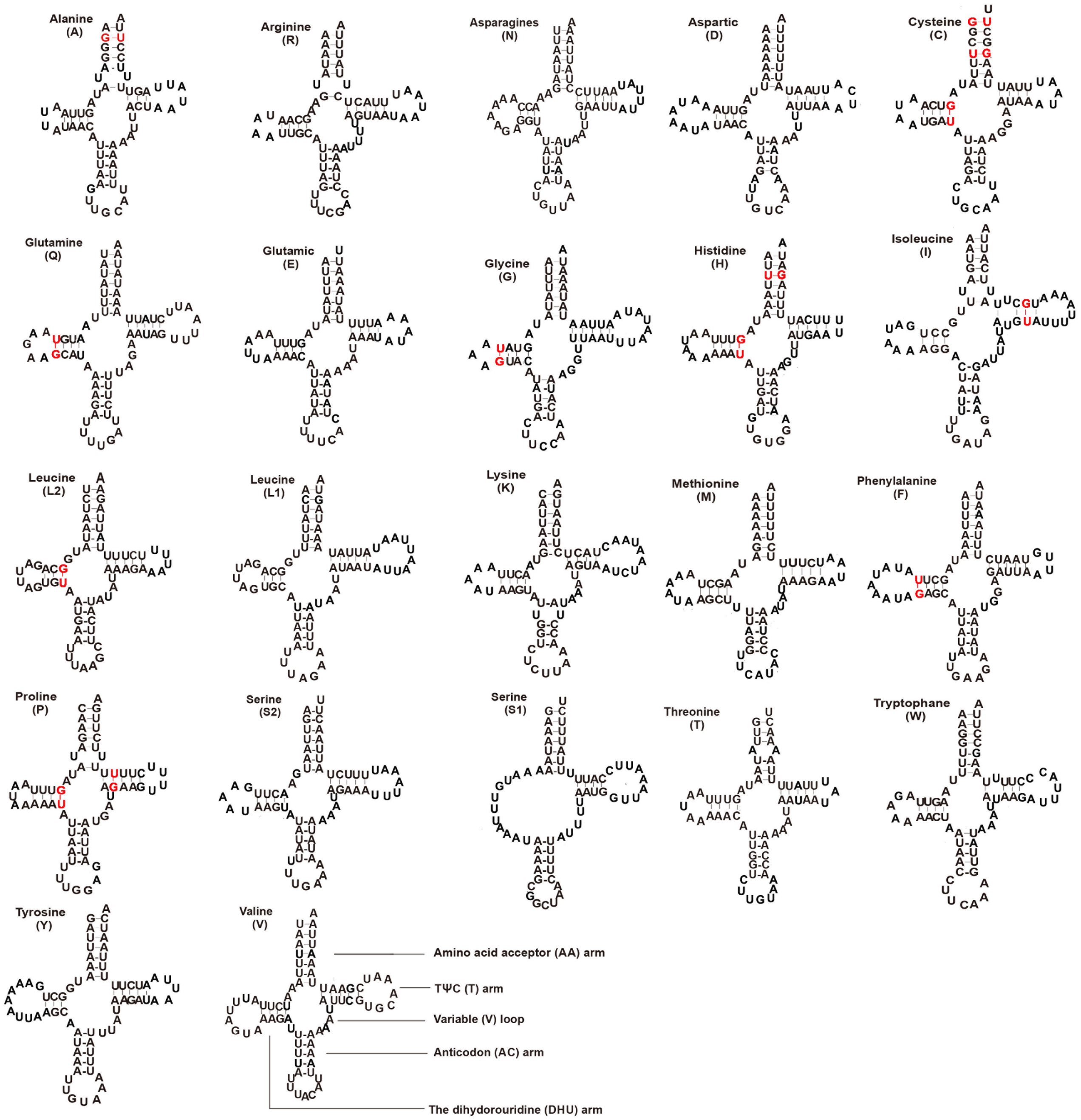
Alanine (A)
Arginine (R)
Asparagines (N)
Aspartic (D)
Cysteine (C)
Glutamine (Q)
Glutamic (E)
Glycine (G)
Histidine (H)
Isoleucine (I)
Leucine (L2)
Leucine (L1)
Lysine (K)
Methionine (M)
Phenylalanine (F)
Proline (P)
Serine (S2)
Serine (S1)
Threonine (T)
Tryptophane (W)
Tyrosine (Y)
Valine (V)
Amino acid acceptor (AA) arm
TΨC (T) arm
Variable (V) loop
Anticodon (AC) arm
The dihydorouridine (DHU) arm
